# Outcomes and eye care knowledge in rhegmatogenous retinal detachment patients with a history of laser refractive surgery for myopia

**DOI:** 10.3389/fpubh.2022.895024

**Published:** 2022-08-11

**Authors:** Chieh Lan, Yi-Hao Chen, Yung-Jen Chen, Jong-Jer Lee, Hsi-Kung Kuo, Pei-Chang Wu

**Affiliations:** ^1^Department of Family Medicine, Kaohsiung Chang Gung Memorial Hospital, Chang Gung University College of Medicine, Taoyuan, Taiwan; ^2^Department of Ophthalmology, Kaohsiung Chang Gung Memorial Hospital, Chang Gung University College of Medicine, Taoyuan, Taiwan

**Keywords:** myopia, retinal detachment, laser refractive surgery, eye care knowledge, scleral buckle, vitrectomy

## Abstract

**Purpose:**

To investigate the surgical outcomes and eye care knowledge of patients with rhegmatogenous retinal detachment (RRD) who had previously undergone laser refractive surgery (LRS) for myopia in a myopia epidemic area.

**Methods:**

This retrospective study included patients with primary RRD who underwent surgery and had a history of LRS for myopia at a tertiary medical center. Data were reviewed from medical charts to analyse the surgical outcomes. Questions about eye care knowledge and attitude toward myopia and LRS were obtained.

**Results:**

A total of 774 patients underwent RRD surgery, among whom 341 (44%) had myopia > −3 dioptres, 66% of whom had high myopia. Thirty eyes of 26 patients had a history of LRS for myopia. The mean age of patients with a history of LRS was significantly lower than that of those without a history of LRS (45.7 ± 2.9 years vs. 53.8 ± 1.0, *p* < 0.001). The mean pre-LRS spherical equivalent was −8.66 ± 0.92 (range: −3.00–−12.00) dioptres. In more than half the patients (*n* = 15, 57.7%), the interval between LRS and RRD was more than 10 years. The primary retinal reattachment rate was only 60%, whereas the final retinal reattachment rate was 93%. The mean final visual acuity (VA) improved from a 20/286 to 20/105 (*p* = 0.006). Linear mixed model analysis showed factors of male sex and macular detachment were significant with poor visual outcome (*p* = 0.046 and 0.008) Eye care knowledge obtained from the 19 RRD patients with history of LRS, 47% of patients (9/19) mistakenly thought that LRS could cure myopia and its complications, and 63% of patients were less willing to visit an ophthalmologist because uncorrected VA improvement after LRS. Eighty-four percent thought that proper knowledge and more education about LRS and myopia for the public are important.

**Conclusion:**

In the RRD patients with a history of LRS for myopia, their age was relative younger. Male sex and macular detachment were associated with poor visual outcome. More education with proper knowledge of LRS, myopia and RRD is recommended for the patients to prevent or early detect the occurrence of RRD.

## Introduction

Myopia prevails globally, and an epidemic of myopia in East and Southeast Asia has been indicated by Morgan et al., reporting the prevalence of 80–90% for myopia in young adults and 10–20% for high myopia (1). This is concomitant with the prevalence of low vision and blindness arising from complications of myopia, such as g cataract formation, retinal detachment from peripheral retinal tears, myopic foveoschisis, macular hole with or without retinal detachment, peripapillary deformation, dome-shaped macula, choroidal/scleral thinning, myopic choroidal neovascularization, and glaucoma (2).

Laser refractive surgery (LRS) is a common surgical procedure which shows a favorable outcome of rapid improvement in uncorrected visual acuity (VA), with minimal postoperative pain and infrequent complications. These advantages further improve the quality of life of myopic patients. However, although LRS can correct refractive errors, it cannot reverse the elongation of the eyeball, as seen in myopia (3–5). Therefore, the risk of complications of an elongated eyeball still exists which may even increase after LRS.

Although the efficacy and predictability of laser-assisted *in situ* keratomileusis (LASIK) reportedly reduce low to high myopia, LRS may lead to various posterior segment complications (6, 7). Arevalo et al. reported the possibility of vitreoretinal complications after LRS, although serious complications occurring shortly after LRS are infrequent (7). An association between RD and LRS has been suspected, which emphasizes the importance of dilated fundus examination before LASIK. Furthermore, patients with a decrease in VA which is less than expected after LRS should be promptly referred to a vitreoretinal specialist.

Previously, a large telephone survey of 4,026 adults investigating their knowledge of myopia was conducted by the Health Promotion Administration in Taiwan. Seventy percent mistakenly thought that LRS for myopia could prevent the complications of myopia, while 64% did not know that high myopia entailed a high risk of RD and macular degeneration (8). Therefore, proper public health education for myopia, LRS, and RD is important and an emerging issue.

This study aimed to investigate the surgical outcomes and eye care knowledge of patients with rhegmatogenous retinal detachment (RRD) and a history of LRS for myopia.

## Methods

This retrospective cohort study enrolled 774 patients who had undergone vitreoretinal surgery between April 2014 and December 2017 to manage RRD at Kaohsiung Chang Gung Memorial Hospital, Kaohsiung, Taiwan. This study was approved by the Institutional Ethics Review Board and adhered to the tenets of the Declaration of Helsinki. Among the total patients, 26 (30 eyes) had a history of LRS for myopia. Data reviewed from charts, included age, sex, myopia status before LRS, VA at the time of RRD detection, VA after surgical intervention, and follow-up duration after surgery for RRD. Surgical procedures included scleral buckling (SB), and pars plana vitrectomy (PPV) with or without lensectomy, endolaser photocoagulation, internal tamponade, a combination of both techniques, or pneumatic retinopexy with long-duration gases, such as C_3_F_8_ or SF_6_. Success of the initial surgical procedure was defined based on the history of reintervention and the outcome of retinal reattachment. The non- to low-myopia, moderate myopia and high myopia were defined as spherical equivalent refractions lower than−3D,−3D or greater but lower than−6D, and−6D or greater, respectively. Primary success was defined as the retinal reattachment in one operation. Questions about eye care knowledge and attitudes toward myopia and the LRS were answered by the patients. The questions included the following: 1. Do you think the LRS cures myopia and the eyeball becomes normal? 2. Do you regularly follow up after LRS? If yes, was the ocular examination performed with or without pupil dilation? 3. Do you think that it is necessary to provide the public with proper knowledge and more education on myopia, LRS, and RRD?

### Statistical analysis

Snellen VA was converted to the logarithm of the minimum angle of resolution (logMAR) for statistical analyses. VAs of “counting fingers,” “hand motions,” “light perception,” and “no light perception” were assigned logMAR values of 2.3, 2.6, 3.0, and 4.0, respectively (9). We analyzed the demographic and clinical factors of patients with RRD using Student's *t*-test. using linear mixed model with compound symmetry model for analyses involving both eyes, multivariate analysis for the final logMAR VA of these 30 eyes was conducted for variables including age, sex (male = 1, female = 0), laterality of eye (right = 0, left = 1), myopic dioptre (spherical equivalent refraction), previous LRS duration (>10 year = 1, <10 year = 0), initial logMAR VA, macular on/off status (on = 0, off = 1), multiple break status (>2 breaks = 1, 1 break = 0), first surgical type (SB = 1, other = 0). The answer to the questionnaire was yes or no for each question and the result of the proportion was demonstrated for each question. For the questionnaire, the content validity index in each item was 1.0, 1.0 and 0.8, respectively. SPSS Base11.0 software (version 11.0; SPSS, Inc., Chicago, IL, USA) was used. Statistical significance was set at *P* < *0.05*.

## Results

During the study period, 774 patients underwent RRD surgery at our hospital. Among them, 341 (44%) patients had myopia > −3 dioptres, 66% had high myopia (-6 dioptres or greater). Thirty eyes of 26 patients had a history of LRS for myopia; 15 (57.7%) were men and 11 (42.3%) were women (Table 1). The mean age of patients with a history of LRS was significantly lower than that of those without a history of LRS [45.7 ± 2.9 years (range: 33–61 years, *n* = 26) vs. 53.8 ± 1.0 years (range: 15–85 years, *n* = 748), *p* < 0.001], and there was no significant difference in sex (57.7 vs. 64.2%, *p* = 0.507).

**Table 1:** Characteristics and surgical outcomes of rhegmatogenous retinal detachment patients with previous laser refractive surgery for myopia.

**No**.	**Sex, Age**	**Eye**	**Myopia (Diopter)**	**Interval** **LRS-RRD(yrs)**	**Initial BCVA while RRD (logMAR)**	**Final BCVA (logMAR)**	**Macular involved**	**Multiple breaks (≥2)**	**Initial surgery type**	**Surgery again**	**Retina outcome**	**Follow up duration (months)**
1	M, 35	OD	−10	>10	1	1.2	(-)	(-)	SB	(-)	Attach	5
2	M, 44	OS	−8	>10	2.6	1.3	(-)	(+)	SB	(-)	Attach	13
3	M, 35	OD	−8	5–10	2	0.5	(-)	(-)	SB	(-)	Attach	53
4	M, 36	OD	−8	>10	0.5	0	(-)	(-)	SB	(+)	Attach	46
5	F, 38	OS	−10	>10	0.7	0.3	(-)	(+)	SB	(+)	Attach	2
6	F, 55	OS	−10	>10	0.5	0.5	(-)	(-)	SB	(+)	Attach	48
7	F, 50	OD	−11	5–10	1	0.1	(-)	(-)	SB	(-)	Attach	46
8	M, 48	OD	−10	<1	2.6	1.5	(+)	(-)	C3F8	(+)	Attach	47
9	M, 52	OU	−10/−10	>10	0.3/0.7 (OD/OS)	0.2/0.1	(-)/(-)	(-)/(-)	TPPV/SB	(-)/(-)	Attach/Attach	45
10	F, 51	OS	−6.5	>10	1.7	0.2	(+)	(-)	SB	(-)	Attach	24
11	F, 46	OD	−10	>10	2.3	0.5	(+)	(-)	SB+TPPV	(-)	Attach	37
12	F, 41	OS	−6	>10	2.6	0	(+)	(+)	SB+TPPV	(-)	Attach	43
13	M, 51	OD	−9	>10	0.3	0.7	(+)	(-)	SB	(+)	Attach	42
14	F, 45	OD	−5	>10	2	0.4	(-)	(-)	SB	(-)	Attach	27
15	M, 48	OD	−8	5–10	0.7	0.7	(-)	(-)	TPPV	(+)	Attach	29
16	M, 45	OD	−7	>10	1.4	2.3	(+)	(+)	TPPV	(-)	Attach	30
17	F, 33	OD	−8	>10	0	0.1	(-)	(-)	SB	(-)	Attach	24
18	M, 47	OU	−10/−10	>10	0.7/1.7 (OD/OS)	0/2.3	(-)/(+)	(+)/(-)	TPPV/SB+TPPV	(-)/(+)	Attach/Attach	30
19	M, 51	OD	−4.5	>10	1.3	0.4	(-)	(-)	SB	(-)	Attach	38
20	M, 47	OD	−7	>10	1.5	1.7	(+)	(-)	TPPV	(-)	Attach	16
21	F, 61	OU	−12	>10	2/1 (OD/OS)	1.7/1.3	(+)/(+)	(-)	TPPV+SB/TPPV	(+)/(+)	Detach/Attach	24
22	F, 38	OD	−8	>10	1.5	0.3	(-)	(-)	SB	(-)	Attach	6
23	M, 57	OD	−6	>10	1.1	1	(-)	(-)	SB	(+)	Attach	18
24	F, 38	OD	−3	5–10	0.5	0	(+)	(+)	SB	(-)	Attach	10
25	M, 47	OU	−13/−13	>10	0.3/0 (OD/OS)	0.2/0	(-)/(-)	(-)/(-)	SF6/C3F8	(+)/(+)	Attach/Attach	13
26	F, 49	OS	−10	>10	0.2	2	(+)	(-)	SB	(+)	Detach	3

LRS: laser refractive surgery; RRD, rhegmatogenous retinal detachment; BCVA, best corrected visual acuity; SB, scleral buckling; TPPV, trans pars plana vitrectomy; C3F8, perfluoropropane; SF6, sulfur hexafluoride.

### RRD with previous LRS

Among the 30 eyes of RRD with previous LRS, the mean pre-LRS spherical equivalent was −8.66 ± 0.92 (range: −3.00–−12.00) dioptres. In more than half of the patients (*n* = 15, 57.7 %), the interval between LRS and RRD was more than 10 years. In only one patient (3.8%), it was within a year.

The primary retinal reattachment rate was 60%, whereas the final retinal reattachment rate was 93.3%. Six eyes (20%) underwent PPV, 50% of which (*n* = 3) underwent surgery again. Seventeen eyes (56.7%) underwent SB, 35.3% (*n* = 6) of which underwent surgery again. Four (13.3%) eyes were operated with combined PPV and SB, 50% (*n* = 2) of which underwent surgery again. Three eyes (10%) underwent pneumatic retinopexy, all of which underwent surgery again.

Among the eyes of RRD with previous LRS, the macula was involved in 12 eyes (40%). Of these, four, four, three, and one eye underwent trans PPV (TPPV) + SB, SB, TPPV, and pneumatic retinopexy, respectively. Multiple breaks (>2 breaks) were observed in six eyes, one of which underwent surgery following the initial SB. Vision improved in 21 (70%) eyes. The final Snellen VA significantly improved from 20/286 to 20/105, with the logMAR value changing from 1.16 ± 0.28 to 0.71 ± 0.26 (*p* = 0.006).

For the analysis of final logMAR VA, multivariate analysis showed no statistically significance with age, laterality of eye, myopic dioptre, previous LRS duration, initial logMAR VA, multiple breaks status, and first surgical type. Two factors, sex and macular status, were statistically significant (*P* = 0.046 and 0.008, respectively Table 2). Male sex and macula-off status were associated with poor visual outcomes.

**Table 2:** Linear mixed model analysis for factors associated with final logMAR visual acuity in the RRD patients with a history of LRS for myopia.

		**Final logMAR VA**			
***N* = 26 patients (30 eyes)**	**coefficient (β)**	**95% CI**			***P***
Age	0.006	−0.035	~	0.047	0.761
Sex (male = 1, female = 0)	0.581	0.012	~	1.149	0.046*
Eye (right = 0, left = 1)	0.014	−0.670	~	0.697	0.962
SER (myopic dioptre, D)	−0.064	−0.205	~	0.077	0.358
LRS period (>10 yr = 1, <10 yr = 0)	0.200	−0.502	~	0.902	0.558
Initial logMAR VA	0.205	−0.142	~	0.552	0.232
Macula off (on = 0, off = 1)	0.913	0.271	~	1.554	0.008*
Retinal break (>2 breaks = 1, 1 break = 0)	−0.136	−0.862	~	0.590	0.691
First surgery type (SB = 1, other = 0)	0.234	−0.476	~	0.945	0.498

95% CI: confidence interval. ^*^ represents statistically significant. RRD = rhegmatogenous retinal detachment; LRS = laser refractive surgery; VA=visual acuity; VA=visual acuity; SER=spherical equivalent refraction; D: diopter.

### Questionnaire

**Figure 1:**
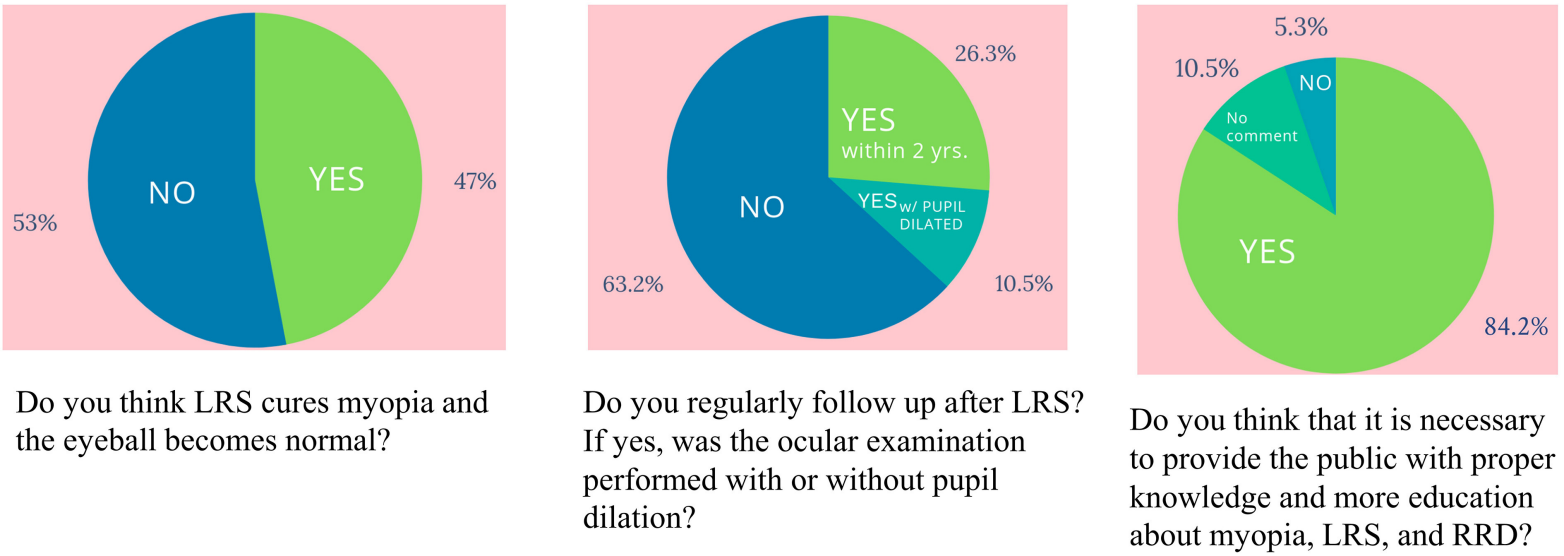
The result of the questionnaire about eye care knowledge and attitude toward myopia and laser refractive surgery.

Eye-care knowledge data were available for 19 patients (Figure 1), 47% of whom thought that their myopia was cured by LRS and that they had healthy eyeballs as normal people. While 64% did not undergo regular follow-up, 36% had regular follow-up within 2 years after LRS, but 71% had no pupil dilation during the retina survey. Furthermore, 63% patients reported that they only went back to the doctor once after LRS, and no further follow-up was requested. Sixteen patients (84.2%) thought necessary to provide the public with proper knowledge and education regarding myopia, LRS, and RRD.

## Discussion

Our study demonstrated that nearly half of the primary RRD patients in this myopia epidemic area of Taiwan were myopic. It also revealed that patients with a history of LRS for myopia developed RRD at a relatively younger age. Furthermore, this is the first report regarding eye care knowledge in RRD patients with a history of LRS for myopia. The outcomes and knowledge were unfavorable in these patients, suggesting the key emerging need for proper public health education regarding myopia and LRS.

In Taiwan, the prevalence of myopia in the older population is much lower than that in the young to middle-aged population, although a substantial population of RRD patients are elderly. A recent study in Taiwan reported that the average age of the incidence of RD was 47.76 ± 0.67 years, with an obvious peak at 50–69 years in both sexes and a secondary peak at 20–29 years in women (10). A study in China reported that the median age of patients with RRD was 51 years, with two peaks of incidence at 60–69 and 20–29 years of age. In our study, 26 patients with a history of LRS had an average age of 45.7 ± 2.9 years while patients with RRD were relatively young. Another study reported a bi-peak pattern in the age distribution of primary RRD that occurred in the third and sixth decades of life (11). In addition, the mean age of the patients with RRD differs according to ethnicity. Chandra et al. reported that South Asians have a younger age of onset and a higher myopic refraction than in Europeans. In this study, the mean age of onset was 58.3 years in European Caucasians and 54.5 years in South Asians, which was consistent with our findings (53.8 ± 1.0 years, *n* = 748) (12).

A pneumatic suction ring is used during the LRS to fix the eyeball, as the vacuum chamber seals against the globe. Intraocular pressure (IOP) may exceed 65 mmHg during resection, and it is both uniform and regular on the cornea of an appropriate diameter. The rapid change in IOP during suction or release of the microkeratome suction ring may mechanically stretch the vitreous base, leading to a higher incidence of posterior vitreous detachment or precipitation of RRD in the eyes (13). In Alrevalo's study, the frequency of RRD after LASIK for myopia was 0.06%, which was much lower than the incidence of RD in myopic eyes in general (0.7–6.0%). They stated that this is probably explained by the fact that most refractive surgery patients undergo preoperative examinations, including dilated indirect fundoscopy with or without scleral depression, and treatment of any retinal lesion predisposing them to the development of RRD before LASIK (14). However, the current study did not present data to support this theory. Only one patient developed RRD within 1 year of LRS. Most patients develop RRD after several years. Other studies have also indicated no direct association between the LRS and RD, as the incidence was mostly low (13–15).

The primary surgical success rate was 60%, which was much lower than the general anatomical success rate reported in other studies (16–19). However, the small sample size (*n* = 30 eyes) in our study should be noted. We therefore performed a power analysis to compare the results of these studies. A Japanese study showed the primary surgical success rate within 6 months was 90.8(2,519/2,775) (18). A German study showed the primary success rate after one operation was 90% (3,420/3,786) (19). An UK study showed the primary success rate with a single procedure was 86.8% (302/348) (16). Another large UK study showed 86.9% (2,958/3,403) (17). To compare with our study, the power was 0.98, 0.98, 0.90, and 0.93, respectively. This means that our RRD patients with a history of LRS had a lower primary success rate. The final reattachment rate of our study was 93.3%. Two other smaller studies showed 100% final reattachment rate for RRD with previous LRS (13, 15). Our study compared to the UK study (97.4%, 339/348) (16), where the power upon analysis was 0.17. This suggests no obvious difference between these two studies in terms of the final reattachment rate.

In the multivariate regression analysis, the poor final visual outcome of RRD patients with previous LRS was associated with male sex and macular detachment. A previous study showed male sex to be a risk factor for pseudophakic RRD (20). The reason remains unknown. Men reportedly have lower utilization of medical care service utilization than women (21, 22). The reason might be speculated to be the severity of RRD due to the delay in seeking medical care. The other factor associated with poor visual outcomes was macular detachment in this study. Previous studies have shown that macula-off RRD negatively affects postoperative BCVA (23, 24). The course of RRD mostly initially develops from a retinal break initially without retinal and macular detachment. Some patients experienced symptoms of floaters or flash sensations at that time. Subsequently, retinal detachment and macular detachment develop accompanied by visual field defects. Early detection and intervention in RRD are important for visual outcomes.

According to the popularity of the LRS for myopia, proper knowledge and education should be spread. Highly myopic eyes are more likely to develop lattice degeneration, retinal breaks, and RRD compared to normal eyes. The average pre-LRS spherical equivalent of our patients was as high as −8.66 ± 0.92 dioptres. Complications of high myopia, including retinal detachment, are characterized by axial length elongation (2). LRS is performed in the cornea to change the refraction but it cannot reverse the elongated axial length. Although their refractive status became relatively low after LRS for myopia, the risk of RRD remained as high as before. Almost half of the patients mistakenly thought that LRS cured myopia, and over half of them did not undergo regular complete fundus examination. As RRD is a vision-threatening disease and myopic RRD patients are relatively young and at a productive age, the better strategy is to prevent regular fundus examination with detect of the associated retinal lesions early-on. In this study, 83% of the patients thought that public awareness of LRS is necessary to encourage regular follow-up. Therefore, proper education including signs of RRD and shared decision making (SDM) for each patient before and after LRS is needed.

Regular dilated retinal examination might be recommended for high myopia even with or without LRS, especially when patients have floater or flash symptoms. It has been reported that evaluation and management of incident acute posterior vitreous detachment (PVD) offer a low cost and favorable cost-utility to minimize the cost and morbidity associated with the development of RRD (25). Furthermore, treatment and prevention of RRD are extremely cost-effective when compared with other treatment for other retinal diseases regardless of treatment modality (26).

The limitations of this study include its retrospective design, small sample size, failure to obtain the pre-LRS fundus status, axial length data, and the type of LRS including PRK and LASIK. Axial myopia is more common and results from an elongated eyeball (27). We obtain the information on the type of previous LRS, although LASIK is much more popular than PRK in Taiwan. The current questionnaire study which n=19 compared with the previous survey *n* > 4,000 is much lower. This small survey was only conducted for RRD patients with a previous LRS history. Seven of them could not be available due to loss of contact. This questionnaire has been designed by retinal specialist, it was not analysis using reliability and validity test. The questionnaire was answered after the RRD surgery and the last question might lead the patient to respond positively. Although showing the sign of the need for education, a large sample study with a questionnaire in patients with LRS is warranted. Another limitation is the mean ± SE in the non-LRS group was unavailable. Only categorical data of non to low-myopia, moderate myopia and high myopia were available in the non-LRS group. In addition, some of the patients were old and had pseudophakia without previous refraction data. Further prospective, longitudinal, and large-scale studies are necessary to determine the cause and effect relationship between RD and LRS.

In the ideal world, all high myopes should preferably have regular ocular and in particular dilated fundus examination; however, this would be difficult in practical reality. We recommend improved public education with regard to 1. LRS only changing refractive error. 2. LRS not altering the risk of complications of high myopia, namely, retinal tears and detachments. 3. Patients with high myopes and LRS should be monitored for acute onset of floaters and flashes, as well as partial loss of visual field or curtain sensation.

In conclusion, a high prevalence of myopia was observed in patients with primary RRD in the area of the myopia epidemic. Patients who developed RD after LRS for myopia were relatively young, and almost half of them mistakenly thought that LRS cured myopia. Therefore, patients should be informed that although LRS corrects the refractive status of myopia, elongated eyeballs still carry a high risk of myopic complications, including RD. More education with proper knowledge of LRS, myopia and RRD is recommended for myopic patients to prevent or detect RRD early.

## Data availability statement

The original contributions presented in the study are included in the article/supplementary material, further inquiries can be directed to the corresponding author.

## Ethics statement

The studies involving human participants were reviewed and approved by Chang Gung Medical Foundation Institutional Review Board. Written informed consent for participation was not required for this study in accordance with the national legislation and the institutional requirements.

## Author contributions

Design the study: P-CW. Data collection and statistics: Y-HC, Y-JC, J-JL, and H-KK. Writing manuscript: CL and P-CW. All authors contributed to the article and approved the submitted version.

## Conflict of interest

The authors declare that the research was conducted in the absence of any commercial or financial relationships that could be construed as a potential conflict of interest.

## Publisher's note

All claims expressed in this article are solely those of the authors and do not necessarily represent those of their affiliated organizations, or those of the publisher, the editors and the reviewers. Any product that may be evaluated in this article, or claim that may be made by its manufacturer, is not guaranteed or endorsed by the publisher.
